# Associations of screen time with symptoms of stress, anxiety, and depression in adolescents

**DOI:** 10.1590/1984-0462/2025/43/2023250

**Published:** 2024-09-09

**Authors:** Maria Carolina Juvêncio Francisquini, Thais Maria de Souza Silva, Géssika Castilho dos Santos, Rodrigo de Oliveira Barbosa, Pedro Henrique Garcia Dias, Ariel Bello Ruiz, Jadson Marcio da Silva, Antonio Stabelini

**Affiliations:** a Universidade Estadual do Norte do Paraná, Jacarezinho, PR, Brazil.; b Universidade Estadual de Londrina, Londrina ,PR, Brazil.

**Keywords:** Mental health, Sedentary behavior, Youth, Psychological illness, Saúde mental, Comportamento sedentário, Jovens, Mal-estar psicológico

## Abstract

**Objective:**

To examine the associations between self-reported screen time and symptoms of stress, anxiety, and depression in adolescents.

**Methods:**

A cross-sectional study was conducted with 982 adolescents aged between 12 and 15 years, enrolled in public schools in Jacarezinho (PR), Brazil. Screen time was assessed by the question “Considering a typical day, how much time do you spend watching TV, playing videogame, using computer or smartphone?” The DASS-21 questionnaire (short form) was used to assess symptoms of depression, anxiety, and stress. Crude and adjusted analyses (age, sex, and maternal level of education) between screen time and mental disorders symptoms were performed using general linear regression models, with Poisson distribution, with significance level at p<0.05.

**Results:**

Higher depressive symptoms were observed in adolescents who reported screen time of 4–6 hours/day (PR 1.35, 95%CI 1.13–1.61) and ≥6 hours/day (PR 1.88, 95%CI 1.62–2.19), compared with their pairs with <2 hours/day. The same was observed for anxiety symptoms with screen time of 4–6 hours/day (PR 1.23, 95%CI 1.04–1.46) and ≥6 hours/day (PR 1.50, 95%CI 1.28–1.77); and stress, with 4–6 hours/day (PR 1.25, 95%CI 1.08–1.44) and ≥6 hours/day (PR 1.49, 95%CI 1.30–1.71), also compared with their pairs with <2 hours/day.

**Conclusions:**

Screen time was positively associated with depressive, anxiety, and stress symptoms in adolescents. Special attention should be given to those who spend more than four hours a day in front of a screen.

## RESUMO

**Objetivo:**

Examinar a associação entre o tempo de tela autorreportado e os sintomas de estresse, ansiedade e depressão em adolescentes.

**Métodos:**

Foi realizado estudo com delineamento transversal com 982 adolescentes entre 12-15 anos, matriculados na rede pública de ensino da cidade de Jacarezinho (PR). Para mensurar o tempo de tela, os adolescentes responderam à pergunta: “Considerando um dia típico, quanto tempo você passa assistindo TV, jogando *videogame*, usando computador ou *smartphone*?”. O questionário DASS-21 (versão curta) foi utilizado para avaliar os sintomas de depressão, ansiedade e estresse. Análises brutas e ajustadas (idade, sexo e nível de escolaridade materna) entre tempo de tela e indicadores de saúde mental foram realizadas por meio do modelo de regressão linear generalizado, com distribuição de Poisson, adotando a significância de p<0,05.

**Resultados:**

Maiores sintomas depressivos foram observados em adolescentes com tempo de tela de 4–6 horas/dia (RP 1,35, IC95% 1,13–1,61) e ≥6 horas/dia (RP 1,88, IC95% 1,62–2,19) quando comparados com seus pares com ≤2 horas/dia. O mesmo foi observado para os sintomas de ansiedade com tempo de tela de 4–6 horas/dia (RP 1,23, IC95% 1,04–1,46) e ≥6 horas/dia (RP 1,50, IC95% 1,28–1,77); e estresse, com 4–6 horas/dia (RP 1,25, IC95% 1,08–1,44) e ≥6 horas/dia (RP 1,49, IC95% 1,30–1,71), também comparados com seus pares com ≤2 horas/dia.

**Conclusões:**

O tempo de tela foi associado positivamente com os sintomas de depressão, ansiedade e estresse em adolescentes. Atenção especial deve ser dada àqueles que passam mais de quatro horas por dia em frente a uma tela.

## INTRODUCTION

Mental disorders represent one of the main challenges faced by health sectors.^1^ It is estimated that approximately 14% of adolescents have a mental disorder, with depression and anxiety being the most common.^2^ Recently, a nationwide study showed that 30% of Brazilian adolescents had symptoms of anxiety, depression, and non-specific somatic complaints.^1^ In addition, modifiable risk factors, such as physical activity and time spent in sedentary behaviors, can contribute to increased rates of common mental disorders in adolescents.^3^

Sedentary behavior is characterized by a set of activities performed in a sitting, lying, or prone position that require an energy expenditure below 1.5 METs.^4^ In this context, researchers’ interest in how screen time (e.g., watching TV, smartphone, tablet and computer use) can affect adolescents’ mental health has been growing. According to the Digital 2023: Global Overview Report,^5^ Brazil is the second country with the highest screen time, especially among young people, drastically exceeding the recommended two hours/day.^6^

Excessive daily screen time has been positively associated with symptoms of hyperactivity/inattention, internalization problems, depression, anxiety, and lower psychological well-being.^7,8^ However, most of the studies assessing the relationship between screen time and mental health in adolescents evaluated only depressive symptoms,^8,9^ while others evaluated excessive screen time using only up to two hours/day as a reference,^7,8,10^ an amount that is easily reached by practically all adolescents today.^11^

Therefore, identifying the amount of daily screen time associated with the risk of these mental disorders in adolescents is important in order to plan specific prevention policies for this age group, especially in middle/low-income countries such as Brazil, where little research has been conducted on this subject. In this sense, the current study aimed to examine the associations between self-reported screen time and symptoms of stress, anxiety, and depression in adolescents. Based on previous evidence in the literature, we hypothesized that higher screen time would be associated with increased stress, anxiety, and depression symptoms.

## METHOD

This study has a cross-sectional design, with baseline data from the ActTeens Program.^12^ It was approved by the Human Research Ethics Committee of the State University of Northern Paraná, Brazil (nº 4.452.513). Secondary public schools in the city of Jacarezinho (PR), Brazil, including students aged between 12 and 15 years (i.e., Grades 8 and 9) were eligible to participate in the study. The schools were recruited through a list provided by the Regional Education Center. Then, emails were sent directly to the eligible schools. Since they expressed interest, a member of the research team met with the school’s agent to explain the study. Inclusion criteria for the schools were being secondary level, having at least one class of the 8^th^ and 9^th^ grades, and physical education classes two times a week. Six schools were considered eligible, and four agreed to participate. The adolescents and their parents/guardians gave written consent to participation. The exclusion criteria were students who had a cognitive deficit reported by teachers or were aged over 15 years. Initially, 1,014 participants were included in the study and, after applying the exclusion criteria, 142 were excluded, resulting in a sample of 982 adolescents.

Trained research assistants conducted all assessments in the school. The data collection period was carried out in two waves: the first wave in March 2022; and the second wave in August 2023, in Jacarezinho (PR), Brazil, during physical education classes. Self-report information was evaluated using specific questionnaires. Two researchers of both sexes conducted anthropometric assessments.

Data collection proceeded as follows:

1) questionnaire with personal information (sex, age, maternal education level), screen time, and symptoms of depression, anxiety, and stress;
2) after completing the questionnaires, students went to a private room for anthropometric assessments.

Screen time was evaluated through a questionnaire developed for this purpose. The adolescents answered the question: “Considering a typical day, how much time do you spend watching TV, playing videogame, using computer or smartphone?”

There were nine categories of answers:

a) none;
b) less than 1h;
c) between 1 and 2h;
d) between 2 and 3h;
e) between 3 and 4h;
f) between 4 and 5h;
g) between 5 and 6h;
h) between 6 and 7h; and
i) more than 7h.

The mid-point time spent in each behavior was computed (e.g., less than 1h was transformed to 0.5 h).^13,14^

The Depression, Anxiety and Stress Scale (DASS-21) short form, which is validated for Brazilian adolescents,^15^ was used to measure symptoms of depression, anxiety, and stress. The DASS-21 is a self-report assessment that comprises 21 questions and contains three subscales scored on a four-point scale each, ranging from 0 (not at all) to 3 (almost always), where the lowest score is better.

Measurements of weight (kg) and height (cm) followed a standardized process and were performed by qualified staff. The weight was measured to the nearest 0.1 kg using a standardized scale, and the height was measured to the nearest 0.1 cm using a stadiometer (Welmy^®^, São Paulo, Brazil). Body mass index (BMI) was calculated by the equation [BMI=weight (kg)/height (m^2^)]. We transformed BMI into age- and sex-specific percentiles, using the Centers for Disease Control and Prevention (CDC) growth charts.^16^

The Kolmogorov-Smirnov test was used to verify data normality. Means and standard deviations (SD) were used to describe the sample. Analysis of the association between screen time and mental health indicators was performed by applying multiple linear regression models, providing standardized coefficients (β) and 95% confidence intervals (95%CI). We created a crude model as well as an adjusted model for sex, age, BMI percentile, and maternal educational level. To test associations between the amount of screen time and mental disorders symptoms, generalized linear regression models, with Poisson distribution (log function) and robust adjustment for variance, were fitted by using the following classification of screen time: <2 hours/day (reference); 2–4 hours/day; 4–6 hours/day; and >6 hours/day, providing prevalence ratios (PR) and 95%CI. Models were adjusted for sex, age, BMI percentile, and maternal educational level. Statistical Package for Social Sciences (SPSS), version 25.0, was used for all analyses, and the significance level was set at p<0.050.

## RESULTS

The study sample comprised 982 adolescents (50.2% females). Table 1 provides the participants’ characteristics. The prevalence of adolescents with overweight/obesity was 29.8%, and 25.9% of their mothers presented elementary school as education level. The mean screen time (ST) found in these adolescents was 3.8 hours/day (SD±2.4), and only 27.6% (271) of them met the recommendation of ≤2 hours/day.^6^ Regarding exceeded ST, 27.7% (272) spent 2–4 hours/day, 20.5% (201) 4–6 hours/day, and 24.2% (238) ≥6 hours/day.

**Table 1:** Characteristics of the sample.

	Complete sample (982)	
	n	Mean±SD
Age (years)	956	13.9±0.9
Weight (kg)	953	57.5±16.2
Height (cm)	943	162.2±8.1
Body mass index (kg/m^2^)	941	21.6±5.2
Stress (score)	981	6.6±5.6
Anxiety (score)	981	5.3±5.3
Depression (score)	979	3.8±3.4
Screen time (minutes/day)	982	228.8±147.3
Nutritional status, n (%)		
Under weight		34 (3.8)
Normal weight		593 (66.4)
Overweight		127 (14.2)
Obese		139 (15.6)
Mother’s educational level, n (%)		
Elementary I incomplete		47 (13.1)
Elementary I complete		46 (12.8)
High school complete		167 (46.5)
Graduated		77 (21.4)
Not informed		22 (6.1)

SD: standard deviation.

When analyzing the associations between screen time and symptoms of mental disorders, we found statistically positive associations with stress (β 0.193, 95%CI 0.003–0.010), depression (β 0.209, 95%CI 0.002–0.007), and anxiety (β 0.112, 95%CI 0.000–0.007) in the crude as well as in the adjusted analyses (Table 2).

**Table 2:** Associations between screen time and symptoms of mental disorders.

	β	Screen time	p-value
		95%CI	
Crude			
Stress	0.21	0.00–0.01	<0.001
Anxiety	0.12	0.00–0.00	0.028
Depression	0.22	0.00–0.00	<0.001
Adjusted			
Stress	0.19	0.00–0.01	<0.001
Anxiety	0.11	0.00–0.00	0.039
Depression	0.20	0.00–0.00	<0.001

CI: Confidence interval. Adjusted for sex, age, body mass index percentile, and maternal education level.

**Figure 1:**
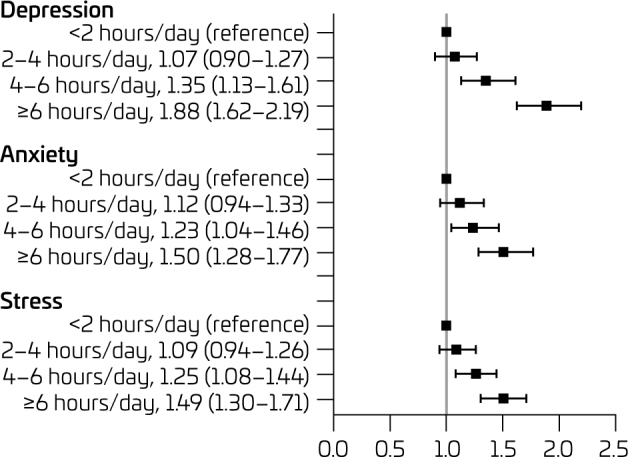
Prevalence ratios between the amount of screen time and symptoms of depression, anxiety, and stress.

Associations between the amount of screen time and symptoms of depression, anxiety, and stress are shown in Figure 1. Higher depressive symptoms were observed in adolescents who expended 4–6 hours/day (PR 1.35, 95%CI 1.13–1.61) and ≥6 hours/day (PR 1.88, 95%CI 1.62–2.19), compared with their pairs with <2 hours/day. Likewise, adolescents who expended >4 hours/day in screen time presented higher symptoms of anxiety [(4–6 hours/day, PR 1.23, 95%CI 1.04–1.46); (≥6 hours/day, PR 1.50, 95%CI 1.28–1.77)] and stress [(4–6 hours/day, PR 1.25, 95%CI 1.08–1.44); (≥6 hours/day, PR 1.49, 95%CI 1.30–1.71)], compared with their pairs with <2 hours/day.

## DISCUSSION

The results of the current study showed that screen time was positively associated with depressive, anxiety, and stress symptoms in adolescents, confirming our initial hypothesis. In addition, we observed that adolescents who spent more than four hours/day on screen time had higher symptoms of common mental disorders compared to those with less than two hours/day.

Adolescents with screen time of 4 to 6 hours/day and more than 6 hours/day had a significant increase of 35% and 88%, respectively, in symptoms of depression compared to those who met the recommendations of up to two hours/day (Figure 1). Our findings on the association between screen time and symptoms of depression in adolescents are in line with previous studies.^3,8,17^ Furthermore, a systematic review found consistent and strong evidence for an association between depressive symptoms and recreational screen time.^18^ The increase in screen-based activities can replace the time spent in interpersonal relationships, leading to social isolation,^19^ and consequently, aggravating depressive symptoms.^20^ The type of content watched during screen time may expose young people to cyberbullying, triggering depressive and anxiety symptoms.^21^ Moreover, screen time spent on social media exposes adolescents to unattainable ideals of beauty, which can have negative consequences about their body image perceptions that can contribute to depressive feelings.^7^

Regarding anxiety symptoms, we observed that adolescents who spent 4 to 6 hours/day and more than 6 hours/day on screen time showed a PR of 23% and 50%, respectively, consistent with the results of studies with Chinese3 and Canadian adolescents.^8^ The content available on social media can contribute to anxiety symptoms,^21^ especially the way how fast-paced media and the type of entertainment affect the emotional and cognitive responses of children and adolescents.^22^ In other words, frequent and excessive exposure to screen time leads individuals to repeatedly change their attention and renew their orientation responses, which increases the neurological excitation,^23^ so they cannot relax for fear of missing out new information, resulting in anxiety symptoms.^24^

Regarding stress symptoms, adolescents who spent 4 to 6 hours/day and more than 6 hours/day on screen time showed an increase in the PR of 25% and 49%, respectively, compared to those who met the recommendations of up to two hours/day. Our results are consistent with prior findings from a study of Canadian adolescents,^25^ in which higher screen time was positively associated with stress symptoms. Increased exposure to screens, especially before bedtime, leads to an expectation of responses to virtual interactions, delaying sleep time, and causing nocturnal awakenings, which can lead to higher symptoms of stress due to sleep deprivation.^26^

In general, the association between excessive screen time and mental health disorders may be explained by direct or indirect mechanisms. Direct pathways can be observed through the content watched on screens, disrupted interpersonal relationships or through direct cognitive effects, creating low emotional stability and impulsivity.^7,21^ Indirect pathways can be observed through certain intermediate factors, such as insufficient sleep, unhealthy eating behaviors, dissatisfaction with body weight, and victimization.^27^ Moreover, excessive screen time can reduce physical activity time, thus limiting the potential benefits of physical activity on mental health.

An important point of the current study is the dose-response pattern found between screen time and symptoms of mental disorders. It was shown that higher screen time increases the odds of depressive, anxiety, and stress symptoms, especially in adolescents who reported spending four hours/day or longer. This finding is extremely worrying, especially among the current generation, as more than 70% of Brazilian adolescents spend more than two hours/day on screen time.^11^

The increase in the prevalence of excessive screen time, mainly in adolescents, may be due to the changes that have occurred in society in recent decades, increasing access to computers, videogames,^8^ and, especially, smartphones,^28^ facilitating internet use in free time. Moreover, our data were assessed after the circumstances imposed by the COVID-19 pandemic. In this context, technology use, more specifically screen use, served as a tool for the maintenance of adolescents’ socialization and school learning.^29^ However, the increase in screen time caused a negative impact associated with enhanced sedentary behavior.^30^

The benefits of physical activity for mental health are well established in the literature.^31^ However, according to Domingues-Montanari,^27^ it does not compensate the adverse effects of screen time. Thus, excessive screen time, whether recreational or not, can cause irreversible damage throughout individuals’ lives.^24^

Some considerations should be made before generalizing the current findings. Firstly, although the data support the hypothesis of a positive association between screen time and mental disorder symptoms, the cross-sectional design of the study prevents us from inferring causality about the relationships. Secondly, screen time was measured using a self-reported questionnaire, which has well-known disadvantages such as memory bias. However, we have used validated questionnaires for this age group, which are easy to use and affordable for large studies. This study also has some strengths. At first, there is an increase of evidence from Brazilian studies on the subject of screen time and mental health outcomes in young people. Moreover, as far as we know, this is the first study to observe a dose-response relationship between excessive screen time and symptoms of mental disorders in Brazilian adolescents.

The high prevalence of adolescents with mental disorders and their consequences has become an important topic for research into risk and protective factors, as a manner to reduce this public health problem. Our findings showed that exposure to more than four hours/day of screen time was associated with higher symptoms of depression, anxiety, and stress, suggesting a worrying risk factor. In this way, the current study makes important contributions, so that specific prevention policies can be planned for this age group to reduce the daily time spent with devices such as television, computer, videogame, and smartphone. Finally, further longitudinal studies are needed to determine whether reducing screen time can have a significant impact on the prevention and treatment of these mental disorders in the pediatric population.

## Data Availability

The database that originated the article is available with the corresponding author.
